# Preliminary report from the World Health Organisation Chest Radiography in Epidemiological Studies project

**DOI:** 10.1007/s00247-017-3834-9

**Published:** 2017-09-21

**Authors:** Nasreen Mahomed, Nicholas Fancourt, John de Campo, Margaret de Campo, Aliu Akano, Thomas Cherian, Olivia G. Cohen, David Greenberg, Stephen Lacey, Neera Kohli, Henrique M. Lederman, Shabir A. Madhi, Veronica Manduku, Eric D. McCollum, Kate Park, Jose Luis Ribo-Aristizabal, Naor Bar-Zeev, Katherine L. O’Brien, Kim Mulholland

**Affiliations:** 1 Department of Radiology, University of the Witwatersrand, Johannesburg, South Africa; 2 Medical Research Council: Respiratory and Meningeal Pathogens Research Unit, University of the Witwatersrand, Johannesburg, South Africa; 3 Johns Hopkins Bloomberg School of Public Health, Baltimore, USA; 4 Murdoch Children’s Research Institute, Melbourne, Australia; 5 Melbourne University, Melbourne, Australia; 6 Department of Radiology National Hospital, Abuja, Nigeria; 7 Medical Research Council, Banjul, Gambia, South Africa; 8 World Health Organization, Geneva, Switzerland; 9 Soroka University Medical Center, Beer-Sheva, Israel; 10 King George Medical University, Lucknow, India; 11 Hospital Sao Paulo, Paulista School of Medicine, Sao Paulo, Brazil; 12 Department of Science and Technology/National Research Foundation: Vaccine Preventable Diseases, University of the Witwatersrand, Johannesburg, South Africa; 13 Kenya Medical Research Institute (KEMRI), Nairobi, Kenya; 14 Johns Hopkins School of Medicine, Eudowood Division of Pediatric Respiratory Sciences, Baltimore, USA; 15 Oxford University Hospitals NHS Foundation Trust, Oxford, UK; 16 Hospital Sant Joan de Déu, Barcelona, Spain; 17 Malawi-Liverpool-Wellcome Trust Clinical Research Programme, College of Medicine, University of Malawi, Blantyre, Malawi; 18 Centre for Global Vaccine Research, University of Liverpool, Liverpool, UK; 19 London School of Hygiene and Tropical Medicine, London, UK

**Keywords:** Bacterial vaccine efficacy trials, Chest radiograph, Child, *Haemophilus influenzae* type b, Pneumonia, *Streptococcus pneumoniae*, World Health Organization defined standardized interpretation

## Abstract

Childhood pneumonia is among the leading infectious causes of mortality in children younger than 5 years of age globally. *Streptococcus pneumoniae* (pneumococcus) is the leading infectious cause of childhood bacterial pneumonia. The diagnosis of childhood pneumonia remains a critical epidemiological task for monitoring vaccine and treatment program effectiveness. The chest radiograph remains the most readily available and common imaging modality to assess childhood pneumonia. In 1997, the World Health Organization Radiology Working Group was established to provide a consensus method for the standardized definition for the interpretation of pediatric frontal chest radiographs, for use in bacterial vaccine efficacy trials in children. The definition was not designed for use in individual patient clinical management because of its emphasis on specificity at the expense of sensitivity. These definitions and endpoint conclusions were published in 2001 and an analysis of observer variation for these conclusions using a reference library of chest radiographs was published in 2005. In response to the technical needs identified through subsequent meetings, the World Health Organization Chest Radiography in Epidemiological Studies (CRES) project was initiated and is designed to be a continuation of the World Health Organization Radiology Working Group. The aims of the World Health Organization CRES project are to clarify the definitions used in the World Health Organization defined standardized interpretation of pediatric chest radiographs in bacterial vaccine impact and pneumonia epidemiological studies, reinforce the focus on reproducible chest radiograph readings, provide training and support with World Health Organization defined standardized interpretation of chest radiographs and develop guidelines and tools for investigators and site staff to assist in obtaining high-quality chest radiographs.

## Introduction

Childhood pneumonia is among the leading infectious causes of mortality in children younger than 5 years of age globally. [1]. *Streptococcus pneumoniae* (pneumococcus) is the leading infectious cause of childhood bacterial pneumonia; it is estimated to have caused 411,000 and 335,000 deaths globally in 2010 and 2015, respectively, in children in this age group [2]. Because bacterial pathogens are most likely to result in death, survival from an episode of pneumonia is dependent on early care-seeking and appropriate triage and treatment, including antibiotics, at the health care facility. Prevention of pneumonia from the two most common bacterial causes of pneumonia, *Streptococcus pneumoniae* and *Haemophilus influenzae* type b, has become routine in low-income countries with the widespread introduction of pneumococcal conjugate vaccines and the *Haemophilus influenzae* type b vaccine in the past decade [3, 4]. Diagnosis of childhood pneumonia remains a critical epidemiological task for monitoring vaccine and treatment program effectiveness.

## World Health Organization standardized chest radiograph interpretation

It is recognized that the chest radiograph has a number of limitations compared to imaging modalities like ultrasound (US) and computerized tomography (CT) of the chest in the diagnosis of childhood pneumonia [5–10]. However, the chest radiograph remains the most readily available and most common imaging modality to assess childhood pneumonia [11]. Due to the limited number of radiologists in low-income countries, clinicians often interpret chest radiographs [11, 12]. In 1997, the World Health Organization Radiology Working Group was established to provide a consensus method to standardize definitions for the interpretation of pediatric frontal chest radiographs only, for use in bacterial vaccine efficacy trials in children. The working group definition was optimized for use as an endpoint in bacterial *Haemophilus influenzae* type b and pneumococcal conjugate vaccines vaccine trials (the so-called “primary endpoint” pneumonia definition), and for determining this spectrum of disease burden from epidemiological studies. The definitions were not designed for use in individual patient clinical management because of their emphasis on specificity at the expense of sensitivity [13].

In bacterial vaccine trials, specificity for measurement of vaccine efficacy is emphasized, with the aim to establish whether the vaccine has activity to prevent pneumococcal or *Haemophilus influenzae* type b pneumonia. If the endpoint lacks specificity, the number of false-positives increases, which reduces the power and precision of the bacterial vaccine trial. Therefore, the World Health Organization standardized chest radiograph definition of primary endpoint pneumonia, which is used as the endpoint in bacterial vaccine trials, compromises on sensitivity, recognizing that certain cases will be missed, but that this does not greatly affect the precision of the vaccine efficacy outcome. The World Health Organization standardized definition of other infiltrates is not used as an endpoint in bacterial vaccine trials [14, 15].

These World Health Organization standardized definitions and endpoint conclusions were published in 2001, and an analysis of observer variation for these conclusions using a reference library of chest radiographs was published in 2005 [13]. The reference chest radiograph library subsequently became a training tool for researchers choosing to use this methodology. During this period, the World Health Organization provided technical support to countries and studies including a centralized arbitration process to resolve discordant interpretations and technical support for sites on chest radiograph methods and equipment.

By 2005, World Health Organization support for standardized chest radiograph interpretation had declined due to resource limitations, despite the increasing application of this tool in a number of epidemiological settings. The applications included pneumonia vaccine efficacy trials, pneumonia vaccine probe studies, malaria efficacy trials, an evaluation of indoor air pollution reduction, pneumonia surveillance activities and pneumonia etiology studies [16–19]. Recognizing a potential drift in the standardized reading and application of the methodology, and seeking a method to maintain the standardized approach to the World Health Organization defined chest radiograph interpretation, this methodology was reviewed at global meetings of technical experts, including the Global Alliance for Vaccines and Immunization GAVI-funded *Haemophilus influenzae* type b Initiative Radiology Workshop in Hanoi, Vietnam, in 2011 [14] and a session at the World Health Organization Pneumococcal Conjugate Vaccine (pneumococcal conjugate vaccines) Impact Evaluation meeting in Geneva, Switzerland, in 2013 [15]. These meetings identified that the methodology achieved good agreement between readers on chest radiograph quality and the presence of primary consolidation (primary endpoint pneumonia), but that some terminology within the definitions required additional clarification [14, 15].

## The World Health Organization Chest Radiography in Epidemiological Studies project

In response to the technical needs identified through these meetings, the World Health Organization CRES project was initiated. It is designed to be a continuation of the World Health Organization Radiology Working Group. The World Health Organization CRES project is a subproject of the pneumococcal conjugate vaccines technical coordination project, which is a collaboration between the Immunization, Vaccines and Biologicals Department of the World Health Organization and the Johns Hopkins University Bloomberg School of Public Health, in Baltimore, Maryland, funded by the Bill and Melinda Gates Foundation. The World Health Organization CRES project is based at the Murdoch Children’s Research Institute in Melbourne, Australia, and is particularly important for countries with planned or ongoing studies to measure the impact of pneumococcal conjugate vaccines, especially in Asia where the uptake of pneumococcal conjugate vaccines has lagged behind that of other regions.

The aims of the World Health Organization CRES project are to clarify the definitions used in the World Health Organization defined standardized interpretation of pediatric chest radiographs in vaccine impact and pneumonia epidemiological studies [13], reinforce the focus on reproducible chest radiograph readings, provide training and support with the standardized interpretation of chest radiographs and develop guidelines and tools for investigators and site staff to assist in obtaining high-quality chest radiographs. The World Health Organization CRES convened a technical working group in June 2016 at the London School of Hygiene and Tropical Medicine to advance these objectives. During the meeting, the World Health Organization CRES Technical Working Group agreed to maintain the existing World Health Organization standardized definitions, but with clarifications to ensure that results from future studies can be bridged back to those of the vaccine trials, impact studies and other pneumonia epidemiological evaluations that used these definitions. The aim of the proposed clarifications is to enhance interobserver agreement of the chest radiograph readings in each of the World Health Organization’s defined categories, and reduce the potential for drift in the application of these definitions within and among chest radiograph readers, both within and among studies over time. The standardized definitions in chest radiograph interpretation would be important for current and future pneumococcal vaccine trials and pneumonia epidemiological studies in children.

**Fig. 1:**
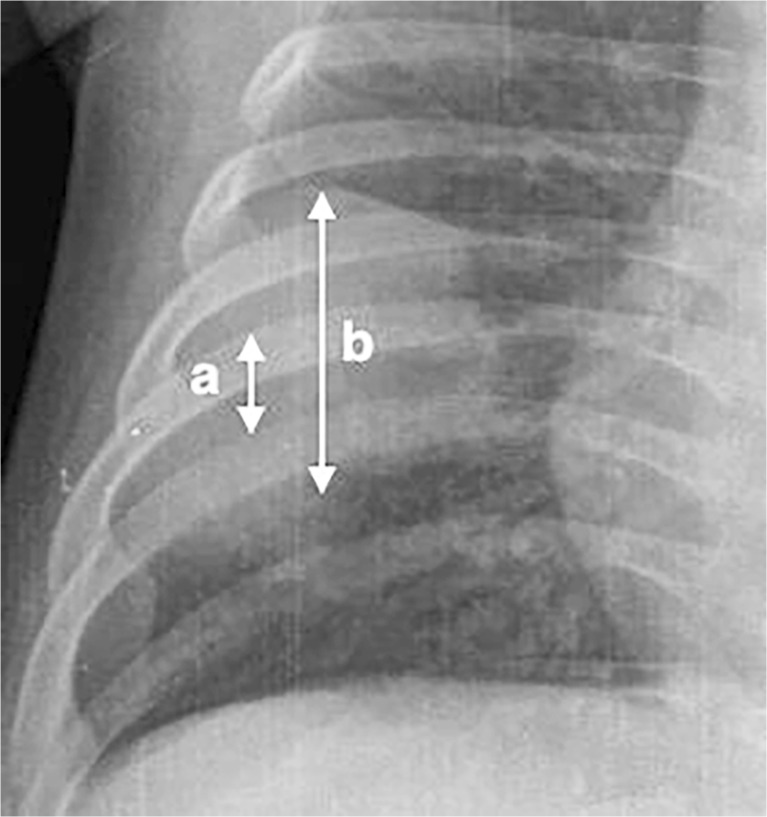
Anteroposterior radiograph cropped to the right hemithorax of a 2 month old male hospitalized with WHO-defined very severe clinical pneumonia and meningitis. Reference measurement of one posterior rib and the adjacent rib space (*double-arrow* **a**). Estimated maximum short-axis diameter of an oval-shaped dense opacity (*double-arrow* **b**)

**Fig. 2:**
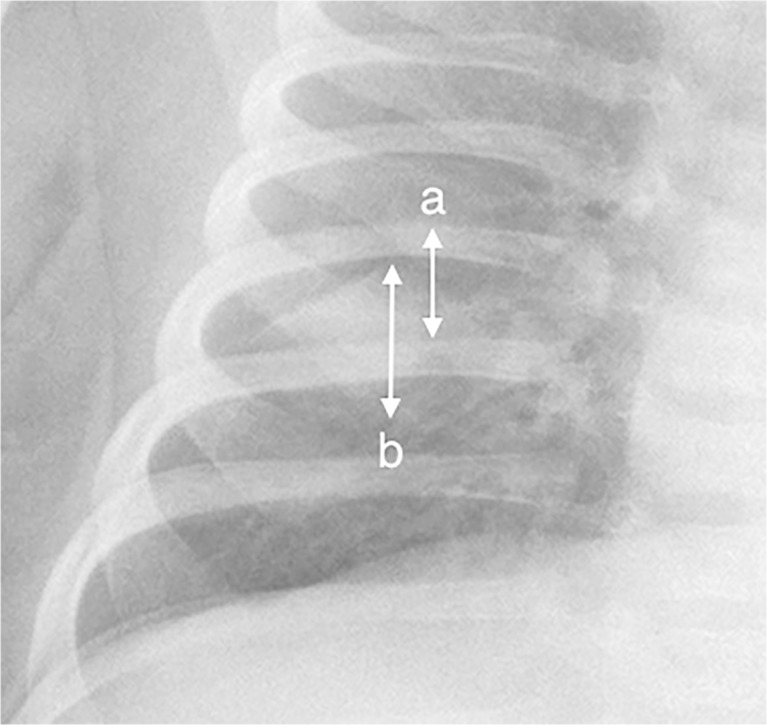
Anteroposterior radiograph cropped to the right hemithorax of a 2 month old female hospitalized with WHO-defined very severe clinical pneumonia. Reference measurement of one posterior rib and the adjacent rib space (*double-arrow* **a**). Estimated maximum short-axis diameter of a wedge-shaped dense opacity (*double-arrow* **b**)

The clarifications to the original World Health Organization standardized chest radiograph interpretation definitions [13] relate to how to quantify the meaning of a portion of a lobe and provide clarity on the inclusion of the silhouette sign to improve reproducibility of chest radiograph reading for primary endpoint pneumonia (Table 1). Here, portion of a lobe means an opacity with the smallest diameter greater or equal to the size of a posterior rib and one adjacent rib space at the same level as the opacity (Fig. 1). Where the opacity is irregular in shape (e.g., wedge-shaped), use the maximum short-axis diameter (the largest diameter perpendicular to the line of maximum diameter of the opacity) (Fig. 2).

**Table 1:** Proposed clarified definitions for World Health Organization defined standardized interpretation of pediatric frontal chest radiographs in pneumonia epidemiological studies

Quality	Uninterpretable	Features of the image are not interpretable with respect to presence or absence of consolidation or pleural effusion without additional images.
	Suboptimal	Features allow interpretation of consolidation and pleural effusion, but not of other infiltrates or findings.
	Adequate	Features allow confident interpretation of consolidation and pleural effusion as well as other infiltrates.
Classification of findings	Significant pathology	Refers specifically to the presence of consolidation, infiltrates or effusion.
	Endpoint consolidation^a^	A dense or confluent opacity that occupies a portion^b^ or whole of a lobe or the entire lung, that may or may not contain air bronchograms^c^.
	Other (non-endpoint) infiltrates	Linear and patchy opacities (interstitial infiltrate) in a lacy pattern, featuring peribronchial thickening and multiple areas of atelectasis; it also includes minor patchy infiltrates that are not of sufficient magnitude to constitute endpoint consolidation, and small areas of atelectasis that in children may be difficult to distinguish from consolidation.
	Pleural effusion	Presence of fluid in the lateral pleural space between the lung and chest wall that is spatially associated with a pulmonary parenchymal infiltrate (including other infiltrate) or has obliterated enough of the hemithorax to obscure any infiltrate; in most cases, this will be seen at the costo-phrenic angle or as a layer of fluid adjacent to the lateral chest wall; this does not include fluid seen in the horizontal or oblique fissures.
Conclusions^d^	Primary endpoint pneumonia	The presence of consolidation or pleural effusion, as defined above.
	Other infiltrate	The presence of other (non-consolidation) infiltrates as defined above in the absence of a pleural effusion.
	No consolidation/infiltrate/effusion	Absence of consolidation, other infiltrates or pleural effusion.

^a^The choice of the term “endpoint” refers to this being the endpoint of interest for trials of bacterial vaccines against pneumonia

^b^“Portion of a lobe” means an opacity with the smallest diameter greater or equal to the size of a posterior rib and one adjacent rib space at the same level as the opacity. Where the opacity is irregular in shape (e.g., wedge-shaped), use the maximum short-axis diameter (the largest diameter perpendicular to the line of maximum diameter of the opacity)

^c^In the presence of any visible adjacent opacity, a silhouette sign, where the length of loss of an anatomical border is greater or equal to the size of a posterior rib and one adjacent rib space at the same level, is considered to indicate consolidation. A silhouette sign of this size without a visible adjacent opacity is considered other infiltrate

^d^Refers to the presence of these conclusions in the opinion of a panel of trained readers using the available World Health Organization defined reference materials and methods

The silhouette sign refers to the absence of depiction of an anatomical soft-tissue border. The silhouette sign results from the juxtaposition of structures of similar radiographic attenuation and this sign actually refers to the absence of a silhouette. It is caused by consolidation and/or atelectasis of the adjacent lung, a large mass or by contiguous pleural fluid [20]. However, it is not always indicative of disease; for example, the absence of a right heart border on chest radiograph can result from pectus excavatum [20].

**Fig. 3:**
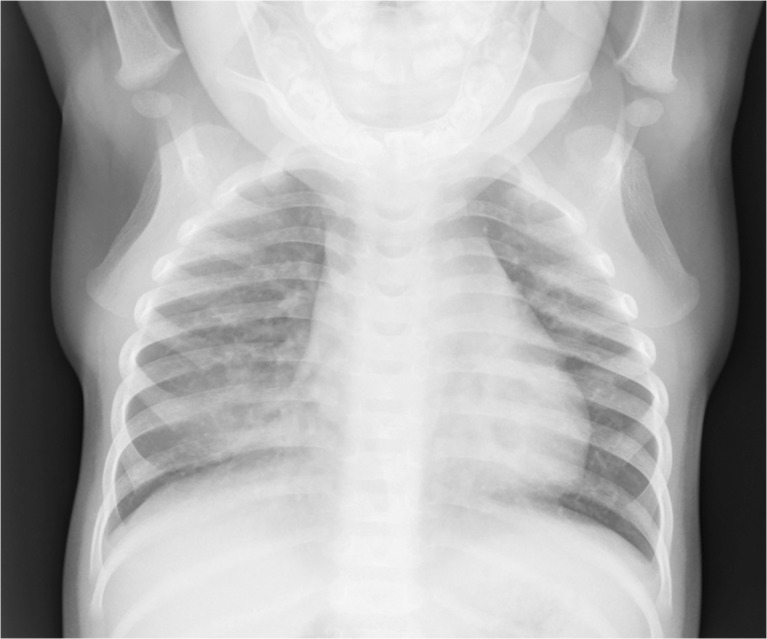
Anteroposterior chest radiograph of an 11 month old male hospitalized with WHO-defined very severe clinical pneumonia demonstrates the silhouette sign with partial loss of the right heart border in the presence of an adjacent opacity, demonstrating endpoint consolidation

A footnote to the original World Health Organization definitions refers to the silhouette sign as follows: “In the presence of any visible adjacent opacity, a silhouette sign, where the length of loss of an anatomical border is greater or equal to the length of a posterior rib and one adjacent rib space at the same level, is considered to indicate of endpoint consolidation. A silhouette sign of this size without a visible adjacent opacity is considered to fulfill the definition of other infiltrate.” This is illustrated in Fig. 3.

An updated set of reference training chest radiographs is being developed under the World Health Organization CRES project. Prior to the June 2016 meeting, the World Health Organization Technical Working Group applied the proposed clarifications to the World Health Organization definitions by interpreting a set of 400 chest radiographs that were not part of the original World Health Organization reference training set (50 from the Gambia Pneumococcal Surveillance Study and 350 from the Pneumonia Etiology in Childhood Research study) and 50 chest radiographs from the original World Health Organization reference training set. These 450 chest radiographs were read by the World Health Organization Technical Working Group members and assigned to one of the three conclusions. Thirteen readers from the World Health Organization Technical Working Group completed all 450 chest radiographs at the time of a data freeze on 20 June 2016. Detailed analysis of these readings showed good correlation between the final reading using the original World Health Organization definitions and those of the new readers using the clarified criteria. As described in the literature using World Health Organization defined standardized chest radiograph definitions [13], the inter-reader agreement for primary endpoint pneumonia is better than the inter-reader agreement for other infiltrates [14, 21, 22]. Using the clarifications to the World Health Organization standardized chest radiograph interpretation, the inter-reader agreement for the 13 readers was best for primary endpoint pneumonia (Cohen kappa = 0.55), which is moderate agreement [23]. A full analysis of these readings for individual chest radiograph findings will be presented in a later publication.

The new set of World Health Organization training chest radiographs are being prepared, emphasizing the need for inclusion of chest radiographs with a high World Health Organization CRES committee reader agreement. The chest radiographs will have comments and annotations added by a subgroup of the World Health Organization CRES project in order to improve their value as a teaching tool. Guidelines for the training and assessment of chest radiograph readers and support for studies in the form of a centralized arbitration process for discordant chest radiograph interpretations are also under development.

The original World Health Organization Radiology Working Group provided standardized definitions on chest radiograph quality. An adequate chest radiograph allows for confident interpretation for primary endpoint pneumonia and other infiltrates. Suboptimal chest radiograph allows for the interpretation of primary endpoint pneumonia but not for other infiltrates. Uninterpretable chest radiograph is not interpretable with respect to the presence or absence of primary endpoint pneumonia or other infiltrates [13] (Table 1). The current World Health Organization CRES project also encompasses the need to optimize both chest radiograph quality and radiation safety for patients and staff. A proposed chest radiograph quality criteria framework is being developed into a set of guidelines for investigators and potential sites with the main objectives being to ensure that chest radiographs are acquired and archived to a specific standard, and that consideration of radiation safety is paramount during the image acquisition process. A checklist with supporting material is under development, which will identify issues to be addressed before, during and after a study involving chest radiographs.

## Conclusion

The World Health Organization CRES project aims to support investigators using the World Health Organization standardized methodology for interpreting pediatric chest radiographs for vaccine efficacy and pneumonia epidemiological studies, including providing clarifications to the original World Health Organization defined standardized chest radiograph interpretation, an updated reference training set of chest radiographs, resources for the training and assessment of readers, guidance on chest radiograph quality and safety, updated reference publications and a centralized arbitration process for the resolution of chest radiographs with discordant interpretations. The final World Health Organization materials and guidance are expected to be published and available in 2017.
